# Acoustic estimation of voice roughness

**DOI:** 10.3758/s13414-025-03060-3

**Published:** 2025-04-28

**Authors:** Andrey Anikin

**Affiliations:** Division of Cognitive Science, Department of Philosophy, Lund University, Box 192, SE- 221 00 Lund, Sweden

**Keywords:** Roughness, Voice perception, Acoustic analysis, Modulation spectrum, Dissonance

## Abstract

Roughness is a perceptual characteristic of sound that was first applied to musical consonance and dissonance, but it is increasingly recognized as a central aspect of voice quality in human and animal communication. It may be particularly important for asserting social dominance or attracting attention in urgent signals such as screams. To ensure that the results of roughness research are valid and consistent across studies, we need standard methodology for measuring it. I review the literature on roughness estimation, from classic psychoacoustics to more recent approaches, and present two collections of 602 human vocal samples whose roughness was rated by 162 listeners in perceptual experiments. Two algorithms for estimating roughness acoustically from modulation spectra are then presented and optimized to match the human ratings. One uses a bank of gammatone or Butterworth filters to obtain an auditory spectrogram, and a faster algorithm begins with a conventional spectrogram obtained with Short-Time Fourier transform; both explain ~ 50% of variance in average human ratings per stimulus. The range of modulation frequencies most relevant to roughness perception is [50, 200] Hz; this range can be selected with simple cutoff points or with a lognormal weighting function. Modulation and roughness spectrograms are proposed as visual aids for studying the dynamics of roughness in longer recordings. The described algorithms are implemented in the function *modulationSpectrum()* from the open-source R library *soundgen*. The audio recordings and their ratings are freely available from https://osf.io/gvcpx/ and can be used for benchmarking other algorithms.

**Supplementary information:**

The online version contains supplementary material available at 10.3758/s13414-025-03060-3.

We all agree that a raven’s croak or the sound of snoring are rougher than the clear note of a nightingale or a soprano singer, but what is it about some sounds that makes them perceptually rough, and can we measure this quality objectively? In the first two sections, I introduce auditory roughness as a fundamental psychoacoustic characteristic and explain why vocal roughness, in particular, has emerged as such an important acoustic feature in recent studies of human and animal vocal communication. I then proceed to discuss historical approaches to measuring roughness acoustically and present two algorithms optimized and validated on two large collections of human vocalizations, speech, and singing whose roughness was rated by listeners in perceptual experiments. The paper concludes with practical recommendations for roughness analysis and a discussion of its limitations.

## What is roughness?

The sense of hearing is designed to analyze minute and extremely rapid fluctuations in air pressure, extracting rich information on different time scales simultaneously. Pressure waves reach the inner ear, where they cause ripples to spread over the basilar membrane, whose different regions resonate at different frequencies (Fastl & Zwicker, 2006; Kandel et al., 2021). Thus, the inner ear effectively performs real-time frequency analysis not unlike a Fourier transform, separating natural sounds into frequency components. However, the analogy is imperfect because, unlike the Fourier transform, cochlear filters are nonlinear: Sinusoidal pressure oscillations are transformed into spike trains that cannot have negative rates (Joris et al., 2004). Thus, the signals that the auditory nerve carries from the inner ear to the brain are essentially *envelopes* of cochlear bandpass filters, and the temporal modulation of these envelopes directly influences perception. For example, a sound that consists of two pure tones at 100 and 110 Hz carries no acoustic energy at 10 Hz, but its envelope, and the firing patterns in the auditory nerve, contains amplitude modulation (beats) at 10 Hz, which is very salient perceptually. In other words, it is a fundamental property of audition to encode modulation within each range of frequencies that activate the same receptor—or, more precisely, the same ganglion cell, which receives input from several inner hair cells (Kandel et al., 2021). These frequency ranges are known as critical bands (Fastl & Zwicker, 2006). As in the example above, two pure tones with frequencies *f1* and *f2* are perceived as a single tone with fluctuating loudness if *f2–f1* is smaller than the critical bandwidth, or as two separate tones with constant loudness if *f2* and *f1* fall into different critical bands (Vassilakis & Kendall, 2010). This is because two tones close in frequency activate the same receptor, and the resulting envelope contains beats caused by interference between these two waves; *f2–f1* is in this case the frequency of beats in the envelope—the modulation frequency.

Sounds are perceived as rough if the envelopes of cochlear filters contain modulation frequencies between about 15 and 300 Hz (Fastl & Zwicker, 2006; Plomp & Steeneken, 1968; Terhardt, 1974a), although the precise range and frequency of peak roughness depend on the stimulus. Modulations slower than 15–20 Hz are perceived as individual fluctuations or acoustic events, and fast modulations with rates over 200–300 Hz merge into a sensation of pitch. While this seems simple enough, the neurological mechanisms of auditory processing are very complex and still imperfectly understood, making it surprisingly challenging to bridge the gap between objective acoustic characteristics of sound and its perceived qualities, including roughness. A key computational feature of auditory processing is that signal frequency can be encoded with a rate-place code or by phase-locking neuronal spikes to the signal (Joris et al., 2004; Kandel et al., 2021). If neurons are arranged in tonotopic arrays, in which every neuron preferentially responds to a narrow range of frequencies, the average rate of firing conveys information about the stimulus frequency. Tonotopic arrays are found from the cochlea to the cortex and contribute to the sensation of pitch as well as modulation (Joris et al., 2004; Plack et al., 2014). Information in rate coding is conveyed by the identity and average firing rate of a neuron, not the timing of each spike. In contrast, if neuronal spikes are synchronized with the signal, the rhythm of firing is also informative. The maximum frequency of phase-locking progressively decreases along the auditory pathway, from several kHz in the auditory nerve (depending on the species) to several hundred Hz in the midbrain and thalamus, and it usually remains under 100 Hz in the primary auditory cortex, similarly to what is found in the primary somatosensory and visual cortex (Joris et al., 2004; Kandel et al., 2021). Phase-locking contributes to pitch perception when harmonics are resolved—far enough in frequency to fall into different critical bands—and it is presumably the only available mechanism of pitch perception based on unresolved harmonics (Plack et al., 2014). For instance, although several upper harmonics of a spoken vowel at 100 Hz fall into the same critical bands, the envelope is amplitude-modulated at the rate of the fundamental frequency (100 Hz). The sensation of pitch at 100 Hz persists if there is no acoustic energy at 100 Hz (missing fundamental), and even if there is no harmonic signal at all, but only broadband noise amplitude-modulated at 100 Hz (residue pitch) because envelopes in several critical bands are phase-locked to the same modulation frequency (Joris et al., 2004; Plack et al., 2014).

Thus, rapid envelope fluctuations in critical bands contribute to the perception of both pitch and roughness, with some overlap in frequency between the two. Of note, it makes no difference what causes the envelope to fluctuate: It can be beats between nearby harmonics within the same critical band (e.g., in musical intervals or overlapping voices; Di Stefano et al., 2022; Terhardt, 1984), amplitude or frequency modulation of the entire sound (e.g., in dysphonic voices, harsh roars, or bird trills with very rapid vibrato), or broadband noise (e.g., in breathy voices or vocalizations with deterministic chaos; Anikin et al., 2021). Envelope fluctuations do not need to be regular to induce the sensation of roughness: Unmodulated broadband noise still sounds rough because envelopes in critical bands drift randomly at all frequencies, including those that sound rough (Fastl & Zwicker, 2006).

The neurological substrates for roughness processing along the ascending pathway from the cochlea to the auditory cortex remain unclear; just as in the case of pitch (Plack et al., 2014), multiple brain regions and computational mechanisms are likely to be involved (Joris et al., 2004). A well-known hypothesis is that the neural signature of roughness is phase-locking to low-frequency periodicities in the cortex (Arnal et al., 2019; Fishman et al., 2000). This forceful synchronization of large-scale cortical networks at a rather high firing rate may be the reason why rough sounds are often perceived as aversive (Arnal & Noemi, 2025). This is in line with the general principle that there are qualitative transitions in subjective experience with increasing rates of sensory events, which has clear parallels in visual and haptic modalities (Di Stefano & Spence, 2022). As frequency exceeds the rate at which successive stimuli are perceived as distinct events (known as the critical segregation rate, which is about 12 Hz for audition; Jurado et al., 2021), the sensation of individual events gives way to visual flicker or acoustic flutter/roughness, and finally to a more continuous sensation such as pitch.

The cortical phase-locking account of roughness is attractive due to its simplicity, but it faces some problems. It is unclear how roughness relates to acoustic flutter, which is represented in the primary auditory cortex by a subpopulation of neurons that synchronize their firing to each acoustic event in the range of about 10–45 Hz (Bendor & Wang, 2007). Roughness and flutter are sometimes equated (Arnal et al., 2019) and treated as a neurological analog to the flicker-fusion transition in vision, which occurs at about 30–50 Hz (Jurado et al., 2021). However, the upper range of reported roughness perception at 200–300 Hz is rather high from this perspective: Despite some evidence of phase-locking in macaques up to 300 Hz (Fishman et al., 2000), it is unusual for thalamo-cortical circuits to synchronize beyond 100 Hz (Joris et al., 2004; Kandel et al., 2021). Furthermore, focusing on the cortex leaves unexplained the role of the very extensive subcortical network for processing envelope modulation, which encompasses much higher modulation frequencies (up to at least 1 kHz in the cochlear ganglion, superior olivary complex, lateral lemniscus, and inferior colliculus) and involves a combination of phase-locking and place-rate coding with tonotopic organization, suggesting one or more filterbanks for extracting the modulation spectrum (Joris et al., 2004). Thus, several cortical and subcortical regions and mechanisms may contribute to the sensation of roughness in parallel with other auditory processing, accounting for the partial overlap of roughness frequencies with both flutter and pitch.

## Why is roughness worth studying?

Auditory roughness is a classic psychoacoustic property that is increasingly used in other fields of inquiry, including research on music, voice pathology, human and animal vocal communication, auditory attention, and cross-modal associations.

### 1. Music and esthetics

Roughness research originated in studies of musical consonance and dissonance that go back at least to Helmholz but remain important to this day (Di Stefano et al., 2022; Terhardt, 1984). Early psychoacoustic research on dissonance was based on the observation that beats between harmonics of two musical tones sound rough and therefore dissonant. Nowadays, there is evidence that both roughness and sharpness negatively affect the perceived consonance of musical intervals, but the effect of roughness is stronger (Eerola & Lahdelma, 2022). Rough-sounding noise was also associated with higher perceived arousal and more negative valence in music, suggesting that roughness affects not only the consonance perception in intervals but also the emotion induced by a musical piece (Blumstein et al., 2012). Furthermore, roughness may be a more universally recognized and esthetically relevant category than consonance (McDermott et al., 2016; Milne et al., 2023). For instance, there is cross-cultural evidence that a dissonant interval is more aversive when both tones are presented in the same ear rather than dichotically. Since beats only appear when both tones are heard in the same ear, this indicates that the unpleasant sensation of dissonance in musical intervals is driven primarily by low-level acoustic roughness (Levelt & Plomp, 1968; McDermott et al., 2016).

### 2. Voice pathology

Roughness is a common feature of pathological human voices, and measuring it accurately can help diagnose voice disorders and quantify their severity (Barsties et al., 2018a; Eddins et al., 2015; Park et al., 2022). For instance, it is well known that vocal instability and roughness can hamper speech intelligibility (Amano-Kusumoto & Hosom, 2011; Anikin et al., 2025; Bender et al., 2004). In addition, increased roughness is consistently named among the most common vocal changes caused by aging, alongside breathiness, strain, instability, and dysphonia (Rojas et al., 2020). This is not limited to human voices: The production of rough-sounding nonlinear vocal phenomena is an indicator of aging in dogs (Marx et al., 2021), and of both aging and sickness in African penguins (Morandi et al., 2025). Acoustic evaluation of roughness could therefore be useful for monitoring vocal health and general physical condition in humans and other animals alike.

### 3. Vocal communication

In humans, rough voices literally sound lower in frequency, enhancing their suitability for intimidating opponents in aggressive contexts (Anikin et al., 2021). Roughness also has a role to play in more peaceful contexts—for example, it signals dominance in conversational laughs (Wood, 2020). All evidence indicates that the association between roughness and dominance or aggression is a fundamental characteristic of vocal interaction between nonhuman animals as well (Morton, 1977). Although auditory perception in most nonhuman species has not been extensively studied, roughness perception seems to be comparable across mammals. For example, cortical responses to rough sounds are very similar in humans and macaques (Fishman et al., 2000). Likewise, seals appear to find the same sounds aversive as do human listeners, being particularly repelled by roughness (Götz & Janik, 2010). On the other hand, bats produce distress calls with amplitude modulation about ten times higher in frequency than the human roughness range, which may or may not be a bat analog of human roughness (Hechavarría et al., 2020). All in all, vocal roughness is a key acoustic characteristic to consider when analyzing nonverbal vocal communication in any species.

### 4. Salience

Another important line of inquiry links roughness with urgency, particularly when signaling danger, and with increasing the potential of calls to attract attention (Arnal et al., 2015, 2019; Arnal & Noemi, 2025; Schwartz & Gouzoules, 2019; Trevor et al., 2020). The key idea is that roughness is inherently salient and unpleasant (Arnal et al., 2019) as well as good for localizing the source of sound (Arnal et al., 2015), so making a vocalization rough would ensure immediate response in an urgent situation. Most evidence is consistent with these claims. For example, auditory salience strongly correlates with the roughness of stimuli with normalized loudness (Zhao et al., 2019), and it can be used as a predictor of salience in natural soundscapes (Huang et al., 2018). As anyone who ever stayed at a dormitory can attest, auditory roughness remains salient during sleep, even when the rough sound is very quiet—such as soft snoring (Legendre et al., 2022). On the other hand, some of the reported facilitatory effects of roughness on sound detection and processing are rather minor (e.g., Taffou et al., 2021), and at least one review concluded that roughness, tonal content, and sharpness are not likely to have a major effect on distraction (Ellermeier & Zimmer, 2014). Stronger evidence of a causal link between roughness and salience would therefore be welcome.

### 5. Cross-modal associations and sound symbolism

Another interesting domain in which roughness is a highly relevant characteristic is cross-modal associations (Spence, 2011). Aristotle hypothesized that amodal properties like roughness, which occur in several sensory modalities, are promising candidates for connecting the senses (Spence & Di Stefano, 2023). An important special case is linguistic iconicity (sound symbolism), which refers to nonarbitrary phonetic properties of words. For instance, words for rough objects tend to contain trilled phonemes, indicating that trilling is a phonetic correlate of haptic roughness (Winter et al., 2022).

In sum, acoustic roughness increasingly figures in a wide variety of theoretical and applied research. What all these domains have in common is that they require a simple and reliable way to measure roughness acoustically. Is there a suitable method for doing so?

## How can roughness be measured?

The obvious gold standard for assessing auditory roughness, which is by definition a subjective perceptual quality, is to ask listeners. There is a tendency to rely on expert judgment for this in voice science and phonetics. For example, Titze (1995) specifically highlighted roughness as a key aspect of voice quality useful for diagnosing voice pathology, and it is one of the most common dimensions used to describe voice quality (Kreiman, 2024) and the severity of dysphonia (Barsties et al., 2018a; Eddins et al., 2015). However, roughness ratings tend to show a lot of individual variation and poor interrater reliability both in natural voices (Kreiman, 2024) and in more controlled stimuli such as synthetic vowels (Bergan & Titze, 2001) or resynthesized human vocalizations (Anikin et al., 2021). Unfortunately, classic psychoacoustic studies of roughness (e.g., Fastl & Zwicker, 2006; Terhardt, 1974a) are often a little opaque about their sample sizes and variability in responses, making interrater reliability a serious concern. Apart from questionable precision of the reported population-level values, we may be missing meaningful individual variation in roughness perception. To take an extreme example, people with hearing loss are more sensitive to roughness at very slow modulation frequencies, down to 3 Hz (Tufts & Molis, 2007).

Despite these difficulties with obtaining ground truth measurements of perceived roughness, there has been a lot of work on estimating it acoustically. Being a psychoacoustic quality, roughness cannot be readily measured from simple spectral descriptives or the musical notation of a chord. In particular, the range of frequencies associated with the sensation of roughness is strongly dependent on the nature of tested stimuli. The sensation of roughness is reported to peak at 75–100 Hz for amplitude-modulated tones (Terhardt, 1974a) and 150 Hz for two-tone stimuli (Plomp & Steeneken, 1968). The foundational book on psychoacoustics (Fastl & Zwicker, 2006) cites a range of 15–300 Hz with a peak at 70 Hz for amplitude-modulated tones with a relatively high carrier frequency. On the other hand, the upper range of rough-sounding amplitude or frequency modulation in voices is occasionally reported to be as low as 70 Hz (Herzel & Reuter, 1996), and amplitude-modulated voices achieve maximum roughness at modulation rates of about 30 to 60 Hz (Eddins et al., 2015; Park et al., 2022). Overall, the range of modulation frequencies perceived as dissonant or rough tends to increase with carrier frequency, reaching ~ 250–300 Hz for carrier frequencies above 2–4 kHz (Fastl & Zwicker, 2006; Fishman et al., 2000). Roughness also depends on the nature of modulation (e.g., frequency modulation sounds rougher than amplitude modulation) and on loudness: Other things being equal, louder stimuli are perceived as rougher (Fastl & Zwicker, 2006). In both pathological and healthy voices, roughness is strongly affected not only by the fundamental frequency but also by irregular phonation such as subharmonics or deterministic chaos (Anikin et al., 2021; Eddins et al., 2015; Park et al., 2022). Even in tonal phonation without dysphonia or nonlinear phenomena, roughness may depend on other aspects of voice quality such as breathiness and glottal pulse shape. In particular, pulse-like phonation in creaky voices or in vocal fry sounds rough because envelopes within multiple critical bands fluctuate at the pulse rate due to the long pauses between two adjacent pulses, making this type of phonation similar to amplitude-modulated noise. In the limiting case, click trains (infinitesimally narrow pulses) sound very rough and aversive at just 40 Hz (Arnal et al., 2019).

Given this complexity, acoustic estimation of roughness requires a physiologically plausible model of the transformation of incoming sound into neural impulses by the auditory periphery and its processing in the brain (Park et al., 2022). In light of the multistage, imperfectly understood neural processing of modulation frequencies, this is not a trivial task. A critical first step is to develop good models of auditory filters in the cochlea. These models are inspired by psychoacoustics (e.g., by studies of tone-in-noise detection), the observed mechanical response of the basilar membrane, or the neural response of the auditory nerve. The most common models of auditory filters use rounded exponential (roex) filters, gammatones, or cascaded recursive filters (Lyon et al., 2010). For example, a popular model uses gammatone filters equally spaced on the Equivalent Rectangular Bandwidth (ERB) scale and with bandwidths either equal to the ERB (Glasberg & Moore, 1990) or proportionate to it (e.g., 1.019 times the ERB; Patterson et al., 1992; Slaney, 1993). The objective of such models is to approximate the frequency response of actual cochlear neurons, but numerous complications arise. First, cochlear responses are nonlinear, so they cannot be adequately captured by linear filters. For instance, bandwidths expand with increasing sound intensity, while the frequency of best response shifts downwards (Shamma et al., 1986). An influential nonlinear model (Shamma et al., 1986; Yang et al., 1992) consists of three stages: a linear basilar membrane with a bank of constant-Q filters, linear fluid–cilia coupling, and an inner hair cell with a saturating nonlinearity due to the transduction process. This model effectively computes an auditory spectrogram using a bank of constant-Q filters, with the bandwidth of each filter equal to about 10% of the central frequency (Elhilali et al., 2003). A mathematically simpler approach, also used in the present study, is to compute the analytic envelope of the output of each filter using the Hilbert transform (Arnal et al., 2015). Another interesting alternative is to model cochlear processing as a traveling wave instead of a bank of discrete filters (Altoè et al., 2021), which could lead to a fundamentally different method of extracting auditory spectrograms and calculating psychoacoustic features.

Some models go beyond auditory periphery and target cortical processing. Chi and colleagues (1999, 2005) presented a mathematical model of central auditory processing, which included progressive loss of temporal dynamics from the periphery to the cortex and emerging sensitivity to combined spectrotemporal features. The initial stage of extracting an auditory spectrogram was broadly similar to earlier models, but with a focus on joint spectrotemporal modulation spectra as a natural way to represent the receptive fields of cortical auditory neurons and neurologically relevant acoustic characteristics of sounds. Spectrotemporal modulation spectra were popularized in publications focusing on speech intelligibility (Elhilali et al., 2003; Elliott & Theunissen, 2009; Greenberg et al., 2003; Greenberg & Kingsbury, 1997; Singh & Theunissen, 2003). Computationally, a modulation spectrum—or a Modulation Power Spectrum (MPS) if it is squared—can be obtained as a two-dimensional (2D) Discrete Fourier Transform (performed as Fast Fourier Transform, FFT) of a spectrogram, which is equivalent to decomposing a spectrogram into a bank of sinusoidal spectrotemporal ripples. Often the spectrogram is log-transformed prior to the 2D FFT to linearize multiplicative modulations (Elliott & Theunissen, 2009), which is a common principle in homomorphic signal processing.

There is nothing about roughness in the original papers on the modulation spectrum. However, it has since been adopted as a method for estimating the roughness of various vocalizations (Arnal et al., 2015). The modulation spectrum represents joint spectrotemporal modulations; if the spectral dimension is integrated out, we are left with temporal modulations, and their normalized average power in some “rough” range of frequencies may be treated as an estimate of perceptual roughness. Following (Arnal et al., 2015), this frequency range is often taken to be 30 to 150 Hz (Arnal et al., 2019; Trevor et al., 2020). Interestingly, no validation of the resulting estimates of roughness has been reported. Judging by the variation in roughness ranges across the psychoacoustic literature, the range of 30 to 150 Hz is reasonable, but not necessarily optimal. The general method of deriving roughness from envelopes of filterbanks is standard in psychoacoustics (Fastl & Zwicker, 2006), and there is some evidence that it generalizes beyond simple stimuli to the analysis of dysphonic human voices (Park et al., 2022). However, there are numerous hidden steps that could have a major impact on the calculated roughness, particularly the method of producing the spectrogram (the number and type of chosen filters, envelopes log-transformed or not, etc.).

In sum, algorithmic calculation of roughness is inspired by a variety of psychoacoustic models. Unfortunately, the mathematical details of these analyses are complex, buried in the small print of supplementary method sections, and justified by reference to a string of previous publications. Tracing this chain back in time, empirical evidence linking algorithms to human ratings of roughness largely comes from mid-twentieth century psychoacoustic studies of musical consonance and of simple artificial stimuli such as pairs of musical tones (Terhardt, 1974b, 1978). In fact, one proposed formula for calculating roughness explicitly represents the signal as pairs of sinusoids (Vassilakis & Kendall, 2010). It is questionable whether this can generalize to more complex natural stimuli such as human or animal voices, leading to revived interest in acoustic estimation of roughness in more realistic voice recordings (Anand, 2023; Awan & Awan, 2020; Barsties et al. 2018b; Eddins et al., 2015; Park et al., 2022). Because the objective is often to diagnose voice pathology, however, many of the proposed methods and acoustic descriptives are speech-specific. Another problem is that the term *roughness* is sometimes applied rather loosely. For instance, one may read that the roughness of elephant rumbles depends on the social context (Soltis et al., 2011), but the acoustic measure actually extracted turns out to be the difference in amplitude between the first and second harmonics, the relation of which to psychoacoustic roughness is unclear.

Given the growing importance of auditory roughness for a number of research fields, the time is ripe to systematize and validate its measurement. The present study aims to take a step in this direction.

## This study

A method of measuring voice roughness from recordings should be as follows:

### 1. Valid

The key requirement is that the algorithm should be perceptually valid for a broad range of realistic sounds, rather than only for combinations of pure tones or highly controlled and static stimuli such as sustained vowels. Apart from being spectrally complex, real-life recordings come with different sampling rates, loudness, background noise, and recording conditions in general. They may also contain pauses and segments with variable roughness, and a good algorithm for calculating roughness should be able both to reproduce the overall perceived roughness of such stimuli and to trace its change over time in a flexible manner (e.g., excluding irrelevant fragments such as unvoiced or quiet frames).

### 2. Easy to use

For applied research, an algorithm for measuring roughness should be user-friendly, taking a recording as input and returning a valid roughness estimate with minimal adjustment of control parameters. For more fundamental research, in contrast, it is helpful to have flexible, open-source algorithms that can easily be customized and shared. Open-source algorithms are also more transparent because we can inspect how a particular result is obtained. Without exception, all the models reviewed above are in-house products that are not freely available to other researchers. Thus, the second biggest gap in roughness research, after the lack of validation experiments, is the absence of open-source solutions.

### 3. Fast

Because large amounts of audio often need to be analyzed in practical applications, the chosen method should be computationally tractable, processing audio at an acceptable rate. Certain approaches, such as massive banks of convolutional filters, may be justified theoretically but simply too slow for most practical applications.

The goal of this paper is to present a solution that satisfies these three desiderata. Specifically, I describe and validate two open-source implementations of roughness analysis in R: (1) an algorithm using auditory spectrograms produced with a bank of bandpass filters and (2) a similarly effective but faster algorithm using ordinary spectrograms produced with Short-Time Fourier Transform (STFT). The proposed method of calculating roughness from spectrograms builds upon the modulation spectrum algorithm proposed by Arnal et al. (2015), but with two modifications designed to make the algorithm both more flexible and more perceptually valid: (1) FFT of each frequency band is performed separately instead of applying a 2D FFT to the spectrogram, and (2) more flexible weighting functions are used instead of simple cutoff points defining the roughness range of modulation frequencies. Both spectrogram extraction details and weighting functions are optimized based on the results of two validation experiments, aiming to approximate human perceptual ratings of the roughness of short extracts of speech, singing, and nonverbal vocalizations.

## Methods

### Stimuli

To ensure better generalizability, two collections of voice stimuli were used in this study, and their roughness was rated by independent samples of listeners. The unaggregated, trial-by-trial ratings of perceived roughness for Corpus 1 were taken directly from a previous study (Anikin et al., 2021), and those for Corpus 2 were collected for the purpose of this study following an identical protocol.

### Corpus 1

The first collection of stimuli consists of 328 resynthesized versions of 82 human nonverbal vocalizations taken from a study of nonlinear vocal phenomena (Anikin et al., 2021). Their duration varies from 260 ms to 2.5 s (total = 5.5 min). Each stimulus was rated on average 15 times on six continuous response scales, including roughness, in a large online experiment by 103 listeners. Specifically, listeners were asked “*Some voices are smooth and clear, others rough and raspy. How rough is this voice?*” and shown images of a smooth pebble and a rough rock for visual reference; responses were collected on a horizontal visual analog scale. The voice quality of the original recordings was manipulated in order to test the perceptual effect of such nonlinearities as subharmonics, deterministic chaos, and rapid amplitude modulation. As a result, these vocalizations varied greatly in their perceived roughness: the median roughness rating per sound ranged from 5.5 to 92.0 on a scale of 0 to 100.

As reported in (Anikin et al., 2021), the interrater agreement was much higher for the three tested psychoacoustic scales (pitch, timbre, and roughness) than for the ecological scales (height, formidability, and aggression). However, the ratings of roughness were still highly variable across listeners, with an intraclass correlation of only .31 (compared with .60 for pitch and .59 for timbre) and a mean Pearson’s correlation between the responses of each participant and aggregated ratings per sound of .74 (compared to .85 for pitch and .85 for timbre). Crucially, there was a noticeable carryover effect of prototype, either because listeners utilized other acoustic cues when estimating roughness or because different versions of the same prototype vocalization were presented in several blocks. For example, despite a clear effect of manipulation on roughness ratings (Anikin et al., 2021), a scream resynthesized as tonal would still be rated as more similar to its “rougher” resynthesized versions than to other prototypes. To account for this prototype effect, a multilevel model was fit to individual roughness ratings (*N* = 5,130) rescaled to range from 0 to 1 and modeled as beta-distributed, with three group-level intercepts: per stimulus (*N* = 328), prototype (*N* = 82), and listener (*N* = 103). The variance of responses per individual was also modeled to account for individual differences in using the response scales. The model was fit with the R package *brms* (Bürkner, 2017) using default priors. Fitted values were then extracted per stimulus while ignoring the prototype intercept—thus, the effect of prototype was effectively subtracted from the estimates.

### Corpus 2

Corpus 2 consists of 274 unmanipulated short recordings of human voices manually selected from a larger collection (Anikin et al., 2023) so as to cover the full gamut of ways in which male and female humans use their voices, from neutral and emotional speech (*N* = 64) to a cappella singing (*N* = 36) and nonverbal vocalizations (*N* = 174). The duration of stimuli ranges from 420 ms to 9.1 s (total = 13 min). Recordings originally longer than that were shortened, retaining a representative section. The perceived roughness of these stimuli was rated in an online perceptual experiment designed to mimic the study in which Corpus 1 was rated (Anikin et al., 2021), as described above. Sixty participants were recruited on the Prolific platform (https://www.prolific.com/) and performed 100 trials each. One participant was excluded because they provided ratings that very poorly correlated with the average responses of other participants (Pearson’s *r* <.2). With the remaining sample size of 59 (26 women, 31 men, two refused to report; mean age ± *SD* = 30.5 ± 8.6, range: 18–53), the roughness of each stimulus was rated on average 22 times.

The median ratings per sound varied from 1.5 to 99.0 on a scale of 0 to 100, indicating that the tested vocal stimuli varied greatly in their perceived roughness. Similarly to Corpus 1, interrater agreement was moderate: intraclass correlation = .47, 95% CI [.42, .52]; median correlation between the responses of each participant and aggregated ratings per sound = .74. Fitted values of perceived roughness per sound were extracted with Bayesian multilevel beta regression fitted to individual responses (*N* = 6,016 trials) in the same manner as above, except that in this case there was no prototype effect to model because all stimuli were unique.

## Acoustic estimation of roughness

Two main approaches were explored, starting with either an auditory spectrogram or a conventional spectrogram produced with STFT. To understand how STFT can be used to measure roughness, it is necessary to briefly recapitulate how it works. The sound is split into a series of overlapping frames, each frame is multiplied by a windowing function to smooth out discontinuities at the ends, a Discrete Fourier Transform is applied to each frame, and the magnitudes of the complex spectra per frame are concatenated, forming a spectrogram. Thus, an STFT spectrogram is a matrix of real numbers that correspond to the magnitude, power (if STFT is squared), or log-magnitude of the spectrum of each windowed analysis frame. The horizontal axis of a spectrogram gives the time of each frame, and the vertical axis the frequency of each frequency bin. Bins with the same frequency form frequency bands.

To be useful for roughness estimation, a spectrogram must possess sufficiently high time resolution to capture beats at least up to 250 Hz—that is, the STFT step should probably not exceed 1,000/250/2 = 2 ms. As for the window length, there are three constraints. First, the bandwidths should be comparable with critical bands. An STFT spectrogram has frequency bands that are linearly spaced and have constant bandwidths (e.g., 50 Hz if window length = 20 ms), so they cannot approximate critical bands over all frequencies, but we do not need that because roughness perception is limited by temporal resolution rather than critical bandwidth in high-frequency bands, bounded by about 250–300 Hz (Fastl & Zwicker, 2006). Thus, we want bandwidths between the lowest relevant critical band (say, 30 Hz for center frequencies under 100 Hz) and about 250–300 Hz, which translates into window lengths of between 1,000/250 = 4 ms and 1,000/30 ~= 33 ms. Second, the STFT window should never be shorter than one period of the fundamental frequency (*f*_0_), otherwise we risk mistaking *f*_0_ for amplitude modulation, even in pure tones (Fig. S1A–B). For human voices with low *f*_0_ values of about 70–100 Hz, this suggests windows > 10 ms. Third, modulation is masked if the STFT window spans more than a couple of periods of the modulation frequency (Fig. S1C–F). Assuming that the fastest modulation frequencies we care about are ~ 250 Hz, the window should ideally be < 8 ms (2 × 1,000/250 = 8), although this is a soft constraint. Obviously, all three of these conditions cannot be satisfied simultaneously, but they roughly define STFT settings that are a priori reasonable for roughness analysis: window length ~ 10–20 ms and step < 2 ms.

The second approach to calculating roughness is to obtain an auditory spectrogram by passing the signal through a bank of bandpass filters and taking Hilbert envelopes of each frequency band, without smoothing or downsampling. The number of filters tested here ranges from one, which corresponds to just the envelope of the sound itself, up to 64. Central frequencies are equally spaced on the ERB, bark, or logarithmic scale ((Eq. 2) Glasberg & Moore, 1990; (Eq. 4) Wang et al., 1992), where:

1 $$ERB=21.4\times \text{log}10\left(1+0.00437\times Hz\right)$$

2 $$Hz=\left({10}^{\left(ERB/21.4\right)}-1\right)/0.00437$$

3 $$bark=6\times asinh\left(Hz/600\right)$$

4 $$Hz=600\times sinh\left(z/6\right)$$

Bandwidths *bw* are either set to a particular number of semitones in constant-Q filters or calculated as a function of the filter’s central frequency *cf*, as (Glasberg & Moore, 1990; Slaney, 1993) follows:

5 $$ERB: bw=24.7\times \left(4.37\times cf/1000+1\right)$$

6 $$Constant-Q: bw=2\times cf\times \left({2}^{\left(s/12\right)}-1\right)/{2}^{\left(s/12+1\right)},$$

where *s = *bandwidth in semitones. Gammatone filters are specified in the time domain as a function of time *t*, as (Slaney, 1993) follows:

7 $$filter={t}^{\left(filter order-1\right)}\times exp\left(-2\times \pi \times bandwith\times t\right)\times cos\left(2\times \pi \times {centerFrequency}\times t\right)$$

Each gammatone is made long enough to include at least 10 periods of its center frequency and convolved with the analyzed sound using FFT convolution. All gammatone filters are of the fourth order, making their shape similar to rounded exponential (roex) filters. Third-order Butterworth filters are implemented recursively using routines from the *signal* library in R (Signal Developers, 2023). Both types of filters are symmetric around the center frequency on a linear frequency scale.

**Fig. 1:**
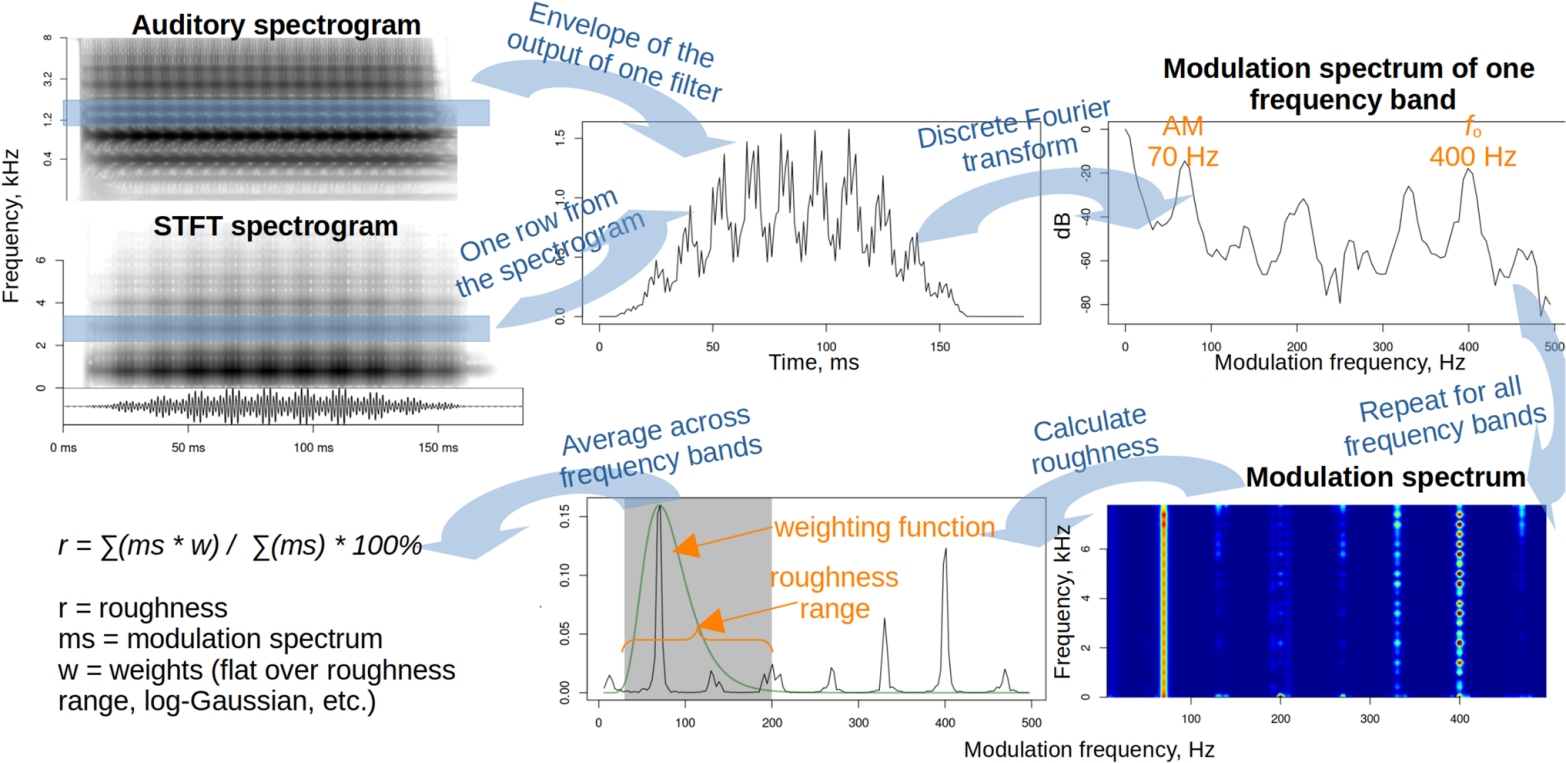
Acoustic estimation of roughness via 1D Fourier transform of each frequency band in the spectrogram. The sound is a synthetic vowel/a/with *f*_o_ = 400 Hz and strong amplitude modulation at 70 Hz. After obtaining either a standard STFT spectrogram or an auditory spectrogram, we take a Discrete Fourier Transform of the envelope of each frequency band and calculate the amount of amplitude modulation in the roughness range. See the text for explanations and *visuals*.*html* for implementation code

After converting the audio to an auditory or STFT spectrogram, the modulation spectrum is calculated by taking either a one-dimensional (1D) FFT of each frequency band or as a two-dimensional (2D) FFT of the entire spectrogram (Fig. 1). If using a 2D transform, the resulting modulation spectrum is folded around the *y-*axis to pool negative and positive temporal modulation frequencies; in all other respects roughness calculation is identical for 1D and 2D versions. Very low modulation frequencies can be discarded as irrelevant for normalization (here, < 1 Hz, although this does not affect the results much—see Table S1, parameter *roughMinFreq*), and then the same weighting function is applied to the temporal modulation in each frequency band, producing a roughness estimate per band:

8 $${r}_{b}=\sum \left({ms}_{f}\times {w}_{f}\right)/\sum \left(ms\right)\times 100\%$$

where *ms*_*f*_ = modulation spectrum of frequency band *f*, *wf* = weighting function for frequency band *f*, and *ms* = modulation spectrum of all frequency bands. The sum of these roughness estimates per band across all frequency bands (optionally, after weighting by the amplitude of each band) gives the roughness of the entire analyzed spectrogram. If the weighting functions are put together in a weight matrix *w*, roughness can be written simply as:

9 $$r=\sum \left(ms\times w\right)/\sum \left(ms\right)\times 100\%$$

Dividing by the sum of the modulation spectrum should not necessary if all analyzed fragments are appropriately normalized: For example, if all inputs have the same energy (root mean square amplitude) and we have their modulation power spectra, comparing ms * w is equivalent to comparing the proportion of energy within the roughness range. However, the inputs may not be normalized, or we may be dealing with longer sounds that are split into shorter fragments with drastically different amplitude levels. This temporal segmentation is controlled by the parameter *amRes*, and it enables the algorithm to return a vector showing how roughness changes over time. The median of these values was found to be a better predictor of human ratings than its mean or maximum value, so it was used for all following analyses and optimizations.

The entire procedure described above is implemented in the function *modulationSpectrum()* from the R package *soundgen* 2.7.1 (Anikin, 2019). This function takes as input a single sound or a folder with multiple audio recordings and returns modulation spectra with roughness estimates per file, as well as more detailed statistics such as roughness in each frequency band of each analyzed sound fragment and the extracted modulation spectra.

### Optimization

The algorithm for acoustic roughness estimation was optimized to reproduce human ratings of the vocal stimuli from both corpora as closely as possible within reasonable constraints on the complexity and runtime of the analysis. Combinatorial explosion made it impossible to permute all parameters passed to *soundgen::modulationSpectrum()* simultaneously. Thus, key settings were analyzed with grid optimization, and the rest were tested by simply changing one parameter at a time. Weighting functions for calculating roughness from modulation spectra were optimized separately from the optimization of the modulation spectra themselves. Population-level estimates of typical perceived roughness were obtained with mixed models applied to raw trial-by-trial responses (see above) and used as ground truth for optimization. Pearson’s correlations between acoustic estimates and human ratings of roughness were calculated separately for the two corpora to ensure that similar performance was obtained with these two very different collections of vocal stimuli. The geometric mean of these two correlations was used as a measure of overall performance, chosen over the arithmetic mean in order to penalize very low correlations in one corpus. When one or both correlations were negative, the following correction was applied:

10 $$\text{gmean}=\left(x,y\left|x<0\space or \space y<0\right.\right)=\sqrt{\left(\left(x+1\right)\times \left(y+1\right)\right)-1}$$

This produces a discontinuity at zero, but near-negative correlations seldom occurred and were never of interest in the present analysis. See supplementary vignettes *optim_audSpec.html*, *optim_STFT.html*, and *optim_final.html* for details on the optimization routines.

## Results

### Estimation of roughness from auditory spectrograms

Perhaps the simplest theoretically grounded method of roughness estimation is to pass the signal through a bank of filters with physiologically realistic shapes and bandwidths, obtain the envelope of the output of each filter, and check how much of its spectrum falls within the range of frequencies typically associated with roughness. With 64 ERB-spaced gammatones (Slaney, 1993), a roughness range of 30–150 Hz (Arnal et al., 2015), and a maximum duration of analyzed fragments of 200 ms (*amRes* = 5), the resulting roughness estimates correlated with human ratings with Pearson’s *r* =.63 for Corpus 1 and *r* =.62 for Corpus 2. Interestingly, switching to the much simpler and faster third-order Butterworth filters with the same ERB bandwidths had little effect on the obtained correlations, leading to *r* = .62 and .67, respectively, for the two corpora. Using these results as an initial benchmark, both types of filters were then optimized by varying their number and bandwidth, and using either the original or log-transformed envelopes as input for the one-dimensional modulation spectrum analysis.

**Fig. 2:**
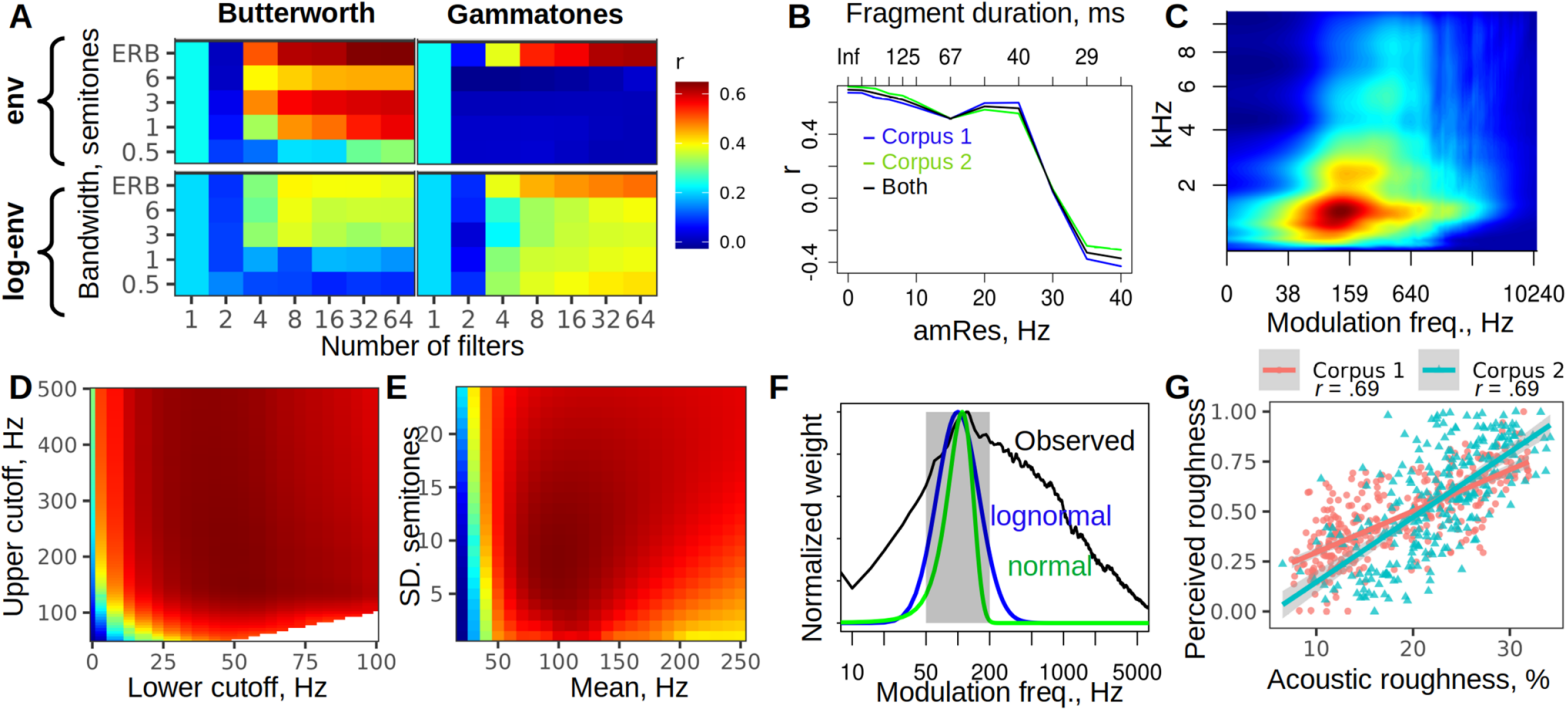
Optimization of roughness estimation via auditory spectrograms. (**A**) Optimization of the number of filters and their bandwidths. Both Butterworth and gammatone filters produce good results with variable ERB bandwidths and without log-transforming the envelopes. At least 16–32 filters are required, with limited extra benefit beyond that number. (**B**) Optimization of the length of analyzed fragments. Splitting a sound into relatively long fragments (~ 200 ms) has a negligible effect on accuracy and provides major gains in processing speed and flexibility. (**C**) Temporal modulation frequencies of about 40 to 300 Hz are more pronounced in rough-sounding stimuli: average modulation spectra of both corpora (*N* = 602 vocalizations) weighted by their *z*-scaled perceived roughness extracted using 64 Butterworth filters with ERB bandwidths. (**D, E**) Optimization of roughness range defined by absolute cutoff frequencies (C) or a lognormal weighting function (D) leads to very similar performance. (**F**) A comparison of roughness weighting functions: Gaussian 110 ± 30 Hz (green), log-Gaussian 100 Hz ± 8 semitones (blue), absolute cutoffs (gray rectangle), and observed mean modulation density plot from the average modulation spectrum of rough sounds in (C). (**G**) Scatterplots of acoustically estimated versus perceived roughness (normalized to range from 0 to 1 for both corpora). (Color figure online)

**Fig. 3:**
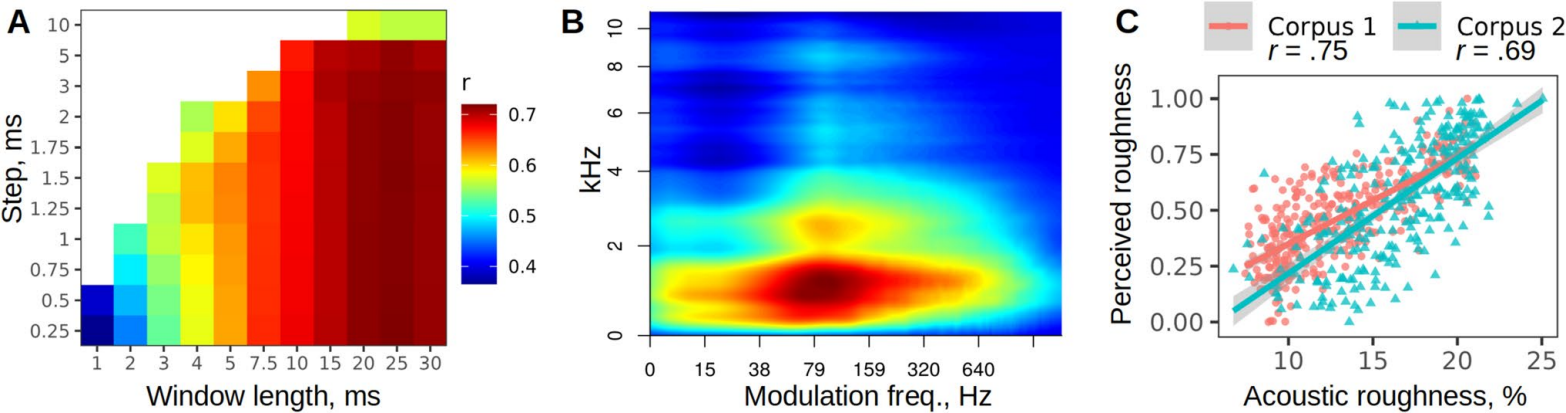
Optimization of roughness estimation via STFT. (**A**) Optimization of window length and step using a *lognormal(100, 8)* weighting function: geometric mean correlation for both corpora. A 25-ms window produces the best results; the step has little effect as long as it is under ~ 3 ms. (**B**) Temporal modulation frequencies of about 40 to 300 Hz are more pronounced in the sounds that humans perceive as rough, especially in the lower frequency bands of the spectrogram: average modulation spectrum of 602 vocalizations weighted by their *z*-scaled perceived roughness extracted with window length = 25, step = 0.25 ms. (**C**) Scatterplots of acoustically estimated vs. perceived roughness (normalized to range from 0 to 1 for both corpora)

The results of optimizing the number of filters and their bandwidths were clear (Fig. 2A) and consistent for both corpora (Fig. S2): the settings used above indeed achieved the best correlation with human ratings. Of note, log-transforming the envelopes had little effect on the achieved correlation for Corpus 1 (best *r* = .62 with 64 gammatone filters), but it was strongly detrimental for Corpus 2 (best *r* = .39 with the same settings; Fig. S2). Reducing the number of filters had a negative effect on accuracy, albeit a minor one: as few as 16 Butterworth filters still produced a correlation of .61 (geometric mean of the correlations with the human ratings of each corpus). The default ERB bandwidths worked well for both gammatone and Butterworth filters. Interestingly, third-order Butterworth filters with a constant-Q bandwidth of about 1–3 semitones (i.e., approximately 6 to 20% of center frequency, Q ~= 5 to 15), whose frequency responses resemble those of gammatones (Fig. S3), performed nearly as well (Fig. 3A, top left subpanel) and achieved correlations of .61 and 67, respectively, when using 32 or 64 filters. Thus, the precise shape of auditory filters may not be very important for the purpose of calculating roughness as long as their bandwidths are comparable to critical bands.

Analyzing long recordings is very slow if gammatones are implemented with FFT convolution, but much faster if each sound is analyzed in shorter fragments (controlled by the *amRes* parameter). This also has the advantage of generating roughness contours per sound instead of just a single estimate regardless of stimulus length. To evaluate the impact of such fragmentation, the effect of *amRes* was tested in a separate optimization round (Fig. 2B). Accuracy did not drop noticeably until the analyzed fragments became shorter than ~ 40 ms (*amRes* > 25). About five roughness measurements per second of audio (*amRes =* 5,200 ms fragments) appears to be a good compromise between precision and the ability to capture changes in roughness over time.

Working with 64 ERB-spaced Butterworth filters with ERB bandwidths and *amRes* = 5, the next round of optimization aimed to verify that 30–150 Hz was indeed the optimal range of roughness frequencies (see *optim_roughRange.html*). Both the inspection of averaged modulation spectra weighted by the roughness rating of each sound (Fig. 2C, F) and the optimization of cutoff frequencies (Fig. 2D) suggest that the optimal range of roughness frequencies is somewhat higher. Changing the roughness range from (30, 150) to (50, 200) Hz improved the geometric mean correlation for the two corpora from.66 to.70. Alternatively, a lognormal weighting function also achieved the same performance (*r* =.70) using a mean of 100 Hz and a standard deviation of eight semitones (Fig. 2E). Frequency-dependent weighting functions offered a negligible improvement (Fig. S4), suggesting that it is acceptable to apply the same weighting function to all frequency bands when calculating roughness. Other tested changes, such as modified filter spacing or bandwidths, also failed to improve the result (Table S1). Interestingly, human ratings were predicted much more accurately without taking the logarithm of either the auditory spectrogram or the modulation spectrum, which goes against the usual practice (e.g., Arnal et al., 2015).

## Estimation of roughness from STFT spectrograms

An alternative to passing the signal through a bank of filters is to start with a conventional STFT spectrogram. As described in Methods, the same algorithm for calculating roughness can then be applied—namely, a 1D or 2D Fourier transform of the spectrogram followed by a weighted average of the temporal modulation spectrum. The *lognormal(100, 8)* weighting function with some reasonable default STFT parameters (here, a 15-ms Hann window and a step of 1 ms) led to roughness estimates that correlated with human ratings with Pearson’s *r* =.75 for Corpus 1 and.66 for Corpus 2. The window length and step were then formally optimized. In line with the theoretically predicted range of STFT settings (see Methods), the best results were achieved using a 25-ms window with the shortest tested step of 0.25 ms: *r* = .75 and .69 for the two corpora, geometric mean = .720 (Fig. 3A). Increasing the step to about 2 ms had a negligible cost in terms of accuracy (geometric mean *r* = .716) and cut the runtime in half. Other variations in the algorithm failed to improve these results. For instance, 2D FFT performed worse, even after a separate optimization of window length and step (Fig. S6). Likewise, performance dropped after log-transforming the spectrogram (*r* = .16 and −.02) or the modulation spectrum (*r* = .30 and .02). The type of window used to produce the STFT spectrogram barely affected the results: Gaussian, Hann (or Hanning), Hamming, or Bartlett windows all produced correlations similar to the second decimal point (*r* = .70).

## Roughness contours in longer recordings

**Fig. 4:**
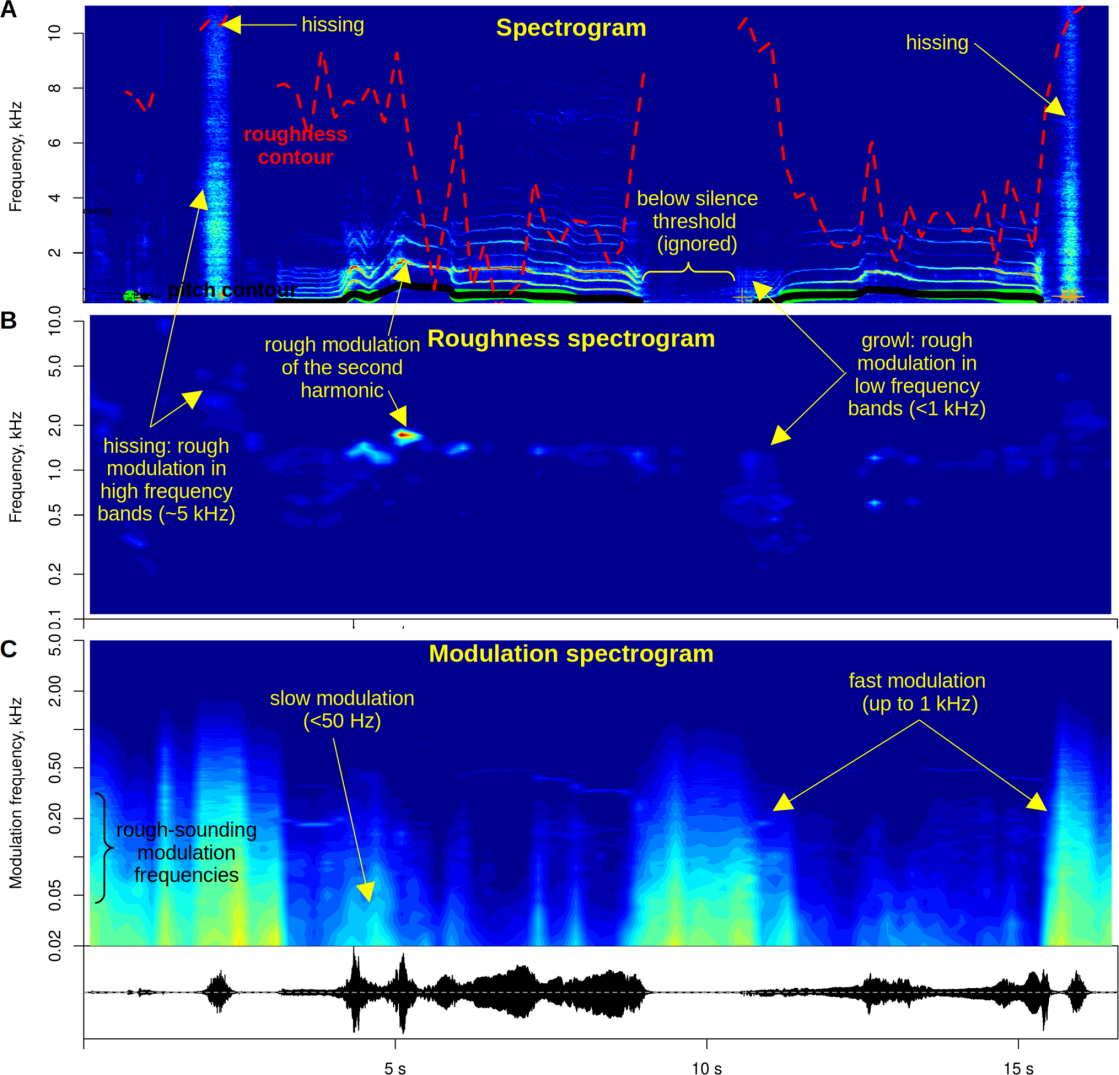
Tracing changes in roughness over time in a longer recording of a human imitating cat vocalizations, from (Anikin et al., 2023). (**A**) An STFT spectrogram overlaid with the roughness contour extracted by the *analyze()* function from *soundgen*. Calling *analyze()* instead of *modulationSpectrum()* makes it straightforward to take both loudness and voicing into account when calculating roughness. (**B-C**) A roughness spectrogram (B) and a modulation spectrogram (C) of the same recording. See text for details, file *cat_imitation.mp4* in supplements for a video representation of these modulation spectra, and vignette *visuals.html* for implementation code

An attractive feature of modulation spectra is that they can be easily and meaningfully averaged across a large number of recordings or fragments, resulting in a typical modulation spectrum of a particular species, call type, or musical genre (Anikin et al., 2023; Elliott & Theunissen, 2009; Singh & Theunissen, 2003). The opposite objective is to trace the changes in roughness over time. Splitting a longer recording into fragments both increases the analysis speed and makes it straightforward to condition roughness on other acoustic features—for example, to give less weight to quiet or voiceless fragments. An easy way to do so is to call the *soundgen* function *analyze()*, which can combine pitch and roughness analysis. For example, we can automatically ignore voiceless frames and exclude very quiet or noisy frames, which would otherwise inflate roughness estimates. This is of little benefit with short, manually prepared and cleaned recordings of the kind used for validation in this study, but essential when working with uncut longer sequences (Fig. 4A).

A useful form of visualization for longer recordings is a *modulation spectrogram*. This term is sometimes used synonymously with *modulation spectrum* to refer to a plot like Figs. 2C and 3B, with modulation frequency along one axis and acoustic frequency along the other, but it seems preferable to reserve the term *spectrogram* for a representation that includes a time dimension. Since a separate modulation spectrum is produced for each analyzed fragment, the output is actually four-dimensional and best visualized in a video format (see *cat_imitation.mp4* in the Supplements), but a spectrogram-like plot can be produced by averaging over either the modulation frequency dimension or the acoustic frequency dimension. The former type was proposed by (Greenberg & Kingsbury, 1997) as a noise-robust representation for speech recognition; the modulation frequencies were collapsed into one value per frequency band by low-pass filtering the modulation spectrum between 0 and 8 Hz. When roughness is the parameter of interest, we can use its value per frequency band and time instead, obtaining a *roughness spectrogram* (Fig. 4B). Alternatively, the modulation spectrum can be averaged across acoustic frequencies in each fragment, resulting in a *modulation spectrogram* (Fig. 4C). Both representations help to visualize modulation in the analyzed sound and to ascertain that the algorithm is working as intended.

## Practical recommendations

A comparison of acoustic roughness estimates with human ratings indicates that auditory spectrograms need at least 16 gammatone or Butterworth filters (the more, the better), whose spacing and bandwidths should match critical bands calculated on a psychoacoustic scale such as ERB or bark. While there are several fast, recursive implementations of gammatones (see Lyon et al., 2010, for review), Butterworth filters are extremely well-understood, widespread, and supported by any software, making them an attractive alternative. Compared with the convolutional implementation of gammatones used here, Butterworth filters were ~ 20 times faster when the entire sound was analyzed at once, although only two times faster when analyzing shorter fragments ~ 200 ms. In practice, any bandpass filters with physiologically realistic bandwidths should produce substantively similar findings. An even simpler and faster approach is to use conventional STFT spectrograms with highly overlapping windowed frames of about 15 to 25 ms. With a step of 2 ms or less, the temporal resolution is sufficient for calculating the temporal modulation spectrum directly from each frequency band in the spectrogram. Although auditory spectrograms are theoretically superior and presumably more generalizable to other stimuli, roughness estimates obtained with STFT and auditory spectrogram were very similar (*r* =.93) and equally valid in relation to human ratings of roughness (*r* ~.70; Table 1).

**Table 1:** Recommended algorithms for calculating perceived voice roughness

Input	Accuracy*	Speed**	R code for measuring with *soundgen 2.7.1*
Auditory spectrogram	.68	3x	modulationSpectrum('../audio/', # path to file or folder specSource ='audSpec', msType ='1D', roughRange = NULL, logSpec = FALSE, logMPS = FALSE, power = 1, roughMinFreq = 1, roughMean = 100, roughSD = 8, amRes = 5, audSpec_pars = list( nFilters = 16, bandwidth = NULL, filterType ='butterworth', yScale ='ERB', dynamicRange = 120))
STFT spectrogram	.72	6x	modulationSpectrum('../audio/', specSource ='STFT', msType ='1D', roughRange = NULL, logSpec = FALSE, logMPS = FALSE, power = 1, roughMinFreq = 1, roughMean = 100, roughSD = 8, amRes = 5, windowLength = 25, step = 2)

^*^ Pearson’s correlations between the acoustic estimates and human ratings of roughness per sound, calculated separately for Corpus 1 and Corpus 2 and averaged with geometric mean.

^**^ Speed relative to real time (seconds of audio per second of machine time) on a consumer laptop running 6 cores in parallel on an Intel i7 - 8550U CPU

Whichever type of spectrogram is chosen, a crucial first step is to plot it for a few stimuli and verify that the algorithm is working correctly—specifically, that some obvious examples of modulation can be visualized in the spectrogram itself, in the modulation spectrum, and in the modulation or roughness spectrogram (Fig. 4). Roughness can then be calculated as a weighted average over a rectangular window from about 50 to 200 Hz, a Gaussian window of about 110 ± 30 Hz, or a log-Gaussian (lognormal) window of about 100 Hz ± 8 semitones. While the results were in this case virtually identical for all three weighting functions, lognormal weighting functions seem safest because they mimic the natural auditory sensitivity to roughness (Fastl & Zwicker, 2006) and should be less dependent on the range of calculated modulation frequencies (e.g., due to differences in sampling rates or STFT step) compared to simple cutoffs.

While 2D FFT was used to generate modulation spectra and calculate roughness in several previous studies (Arnal et al., 2015, 2019; Trevor et al., 2020), slightly better results were obtained here when the spectrum of each frequency band was calculated separately with 1D FFT. An important computational advantage of the 1D method is that multiple short-time spectra can be calculated and averaged instead of applying the FFT to entire envelopes; this is both faster and more robust to noise. Furthermore, when using a 1D FFT, the Y-axis of the resulting modulation spectrum is identical to that of the spectrogram, making it more transparent and convenient for producing a roughness spectrogram (Fig. 4B). A 1D version is also better for implementing frequency-dependent roughness ranges, if required. Overall, however, the algorithm is robust to small changes in the parameters, as can be seen from the broad regions of near-optimal performance in the parameter space (Figs. 2 and 3). In particular, slightly different auditory filters or cutoff frequencies should normally lead to very similar estimates of roughness, and there is large room for adjusting the algorithm depending on the desired speed–accuracy trade-off.

In terms of preparing stimuli for acoustic analysis, no particular normalization is required if roughness is expressed as a proportion of the modulation spectrum. Of course, the subjective perception of roughness does increase with loudness (Fastl & Zwicker, 2006), but this should not greatly affect the relative roughness ratings in perceptual studies as long as the stimuli are normalized prior to playback. Furthermore, changes in the sampling rate between 8 kHz and 44.1 kHz, and therefore in the highest modulation frequencies represented in the envelopes of auditory filters, also have very little effect on acoustic roughness estimates (see *optim_audSpec.html*). In contrast, nearly silent sections of recordings filled with background noise can register as very rough and skew the estimates. This problem can be mitigated by using a silence threshold and/or ignoring very noisy or voiceless frames. The easiest way to do so is to combine roughness and pitch analysis (Fig. 4). Another option is to weight roughness estimates per fragment by the fragment’s loudness or to calculate the total energy within the roughness range of modulation frequencies instead of the proportion of modulation spectrum within the roughness range (i.e., to skip the normalization step in Eqs. 8 and 9).

## Discussion

We are clearly far from the point at which acoustic roughness estimation could obviate expensive perceptual experiments by predicting perceived roughness with sufficient accuracy to answer substantive research questions. Of course, sometimes we have no choice but to rely on acoustic measurements alone—for instance, when we are interested in estimating the roughness of animal vocalizations or need to process banks of recordings that are too large for perceptual experiments. The acceptable level of performance can depend on the application; in this study, acoustic estimates captured approximately 50% of variance in average ratings per stimulus. However, would we arrive at the same substantive conclusions if we relied on acoustics and did not have roughness ratings? Corpus 1 used in this study includes versions of the same vocalizations with manipulated nonlinear vocal phenomena: amplitude modulation, subharmonics, and chaos. While humans rate subharmonics and amplitude modulation as intermediate in roughness, acoustic estimates of roughness place subharmonics closer to regular phonation without nonlinear phenomena (Fig. S7A–B). Systematic differences between perceptual ratings and acoustic estimates are even more apparent in Corpus 2 (Fig. S7C–D). For example, the acoustic roughness of strongly aspirated vocalizations (sighs and grunts) approaches that of roars, whereas listeners do not perceive sighs as rough. This is an example of a situation in which excluding voiceless frames from roughness calculation may be warranted. A more fundamental constraint is that listeners may be mentally “denoising” a recording by ignoring background noise or breathing in their roughness judgments, which is simply not part of the acoustic model. High-level semantic interpretations may further affect roughness ratings by listeners: for example, classical singing in Corpus 2 is rated as less rough than lullabies and chants, whereas this distinction is much less clear-cut based on acoustic analysis (Fig. S7C–D).

In other words, while acoustic estimates of roughness are of course expected to correlate with human ratings, a perfect fit may not be obtainable or even desirable because listeners’ judgments partly reflect top-down expectations (e.g., related to musical genres). This discrepancy may be particularly pronounced in the case of longer and more complex, culturally and contextually loaded stimuli. Furthermore, and in line with previous reports (Anikin et al., 2021; Kreiman, 2024), roughness ratings are very individually variable. As a result, the population average may be far from how each sound is perceived by a particular person in a particular listening setting. As always, caution is required when abstracting from psychoacoustic characteristics of elementary perceptual stimuli to more complex sensory input. Another limitation of the proposed algorithm is that, although it draws inspiration from biology, it is not designed to model the neurophysiology of roughness processing, but rather to obtain valid roughness estimates for a wide variety of vocal stimuli in a conceptually transparent and computationally efficient manner. With these important provisos, the psychoacoustics of roughness perception can clearly guide the analysis of ecologically relevant sounds such as music, human voice, and most likely the calls of other animal species.

## Supplementary information

Below is the link to the electronic supplementary material.

Supplementary file1 (PDF 1947 KB)

## Data Availability

All data and materials are available online (https://osf.io/gvcpx/). None of the experiments was preregistered.

## Code availability

All data and materials are available (https://osf.io/gvcpx/). This includes R code for running all reported analyses. The main code for roughness analysis is included in the open-source R package *soundgen* freely available (https://cran.r-project.org/package=soundgen).
