# Supplementary material for: Acoustic estimation of voice roughness
Source: Atten Percept Psychophys. 2025 Apr 28;87(5):1771–87. doi: 10.3758/s13414-025-03060-3 (PMC12204943; doi:10.3758/s13414-025-03060-3)
Supplement: Supplementary file 1 — Supplementary file1 (PDF 1947 KB) [file 13414_2025_3060_MOESM1_ESM.pdf]

Supplementary figures and tables

Table S1 Optimization of filter banks

	Pearson's *r*		
Arguments passed to *modulationSpectrum()*	Corpus 1	Corpus 2	Both (geom. mean)
Baseline (as in Table 1 / *optim_final.html*)	0.72	0.69	0.7
roughMinFreq = 15	0.68	0.59	0.63
roughMinFreq = 0	0.71	0.67	0.69
msType = "2D"	0.67	0.66	0.67
power = 2	0.67	0.66	0.66
logMPS = TRUE	0.29	-0.17	0.03
nFilters = 128	0.72	0.69	0.71
yScale = "log"	0.72	0.69	0.7
yScale = "bark"	0.73	0.68	0.7

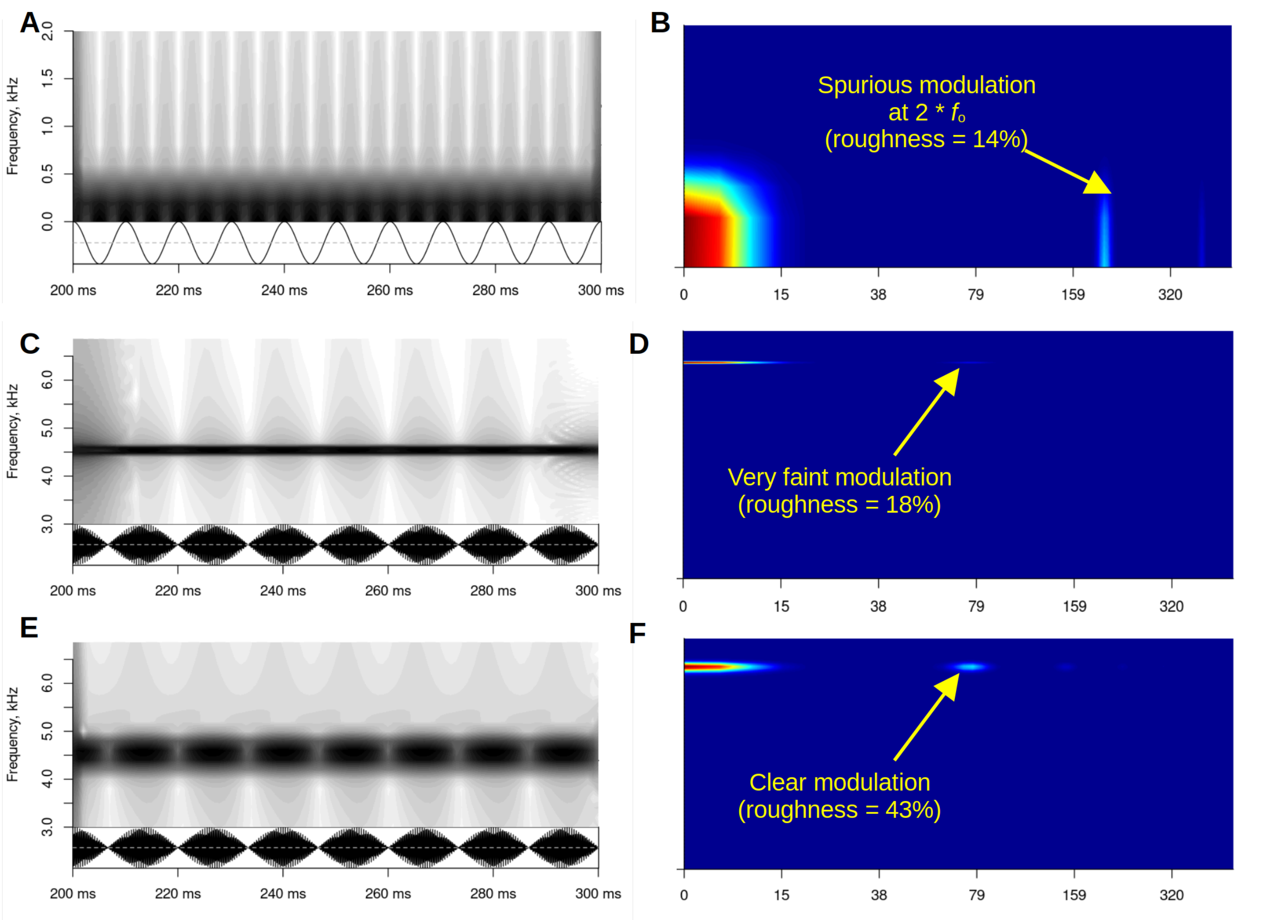

Fig. S1 Selection of STFT window for roughness analysis. (**A, B**) Spectrogram and modulation spectrum of a pure tone at 100 Hz produced with a window length of 25 ms and a step of 1 ms. Alternate STFT frames vary in amplitude, giving the spectrogram a striped appearance; this produces a strong peak at 200 Hz in the modulation spectrum and increases the apparent roughness. (**C-F**) Spectrograms and modulation spectra of a combination of two pure tones at 4500 and 4575 Hz. The beats at f2-f1 = 75 Hz are barely visible in the spectrogram with a window of 25 ms (C-D), but clear with a window of 5 ms (E-F). This has a huge effect on apparent roughness. Auditory spectrograms always have sufficient time resolution and are immune to these problems. See *visuals.html* for more details and code.

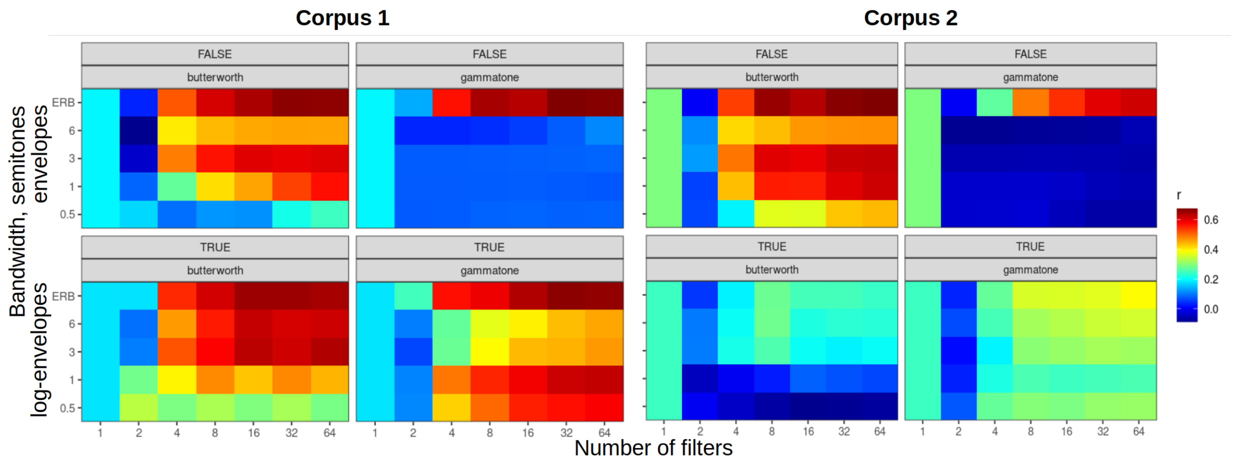

Fig. S2 Optimization of auditory filters: the same as Fig. 2A, but shown separately for Corpus 1 and Corpus 2.

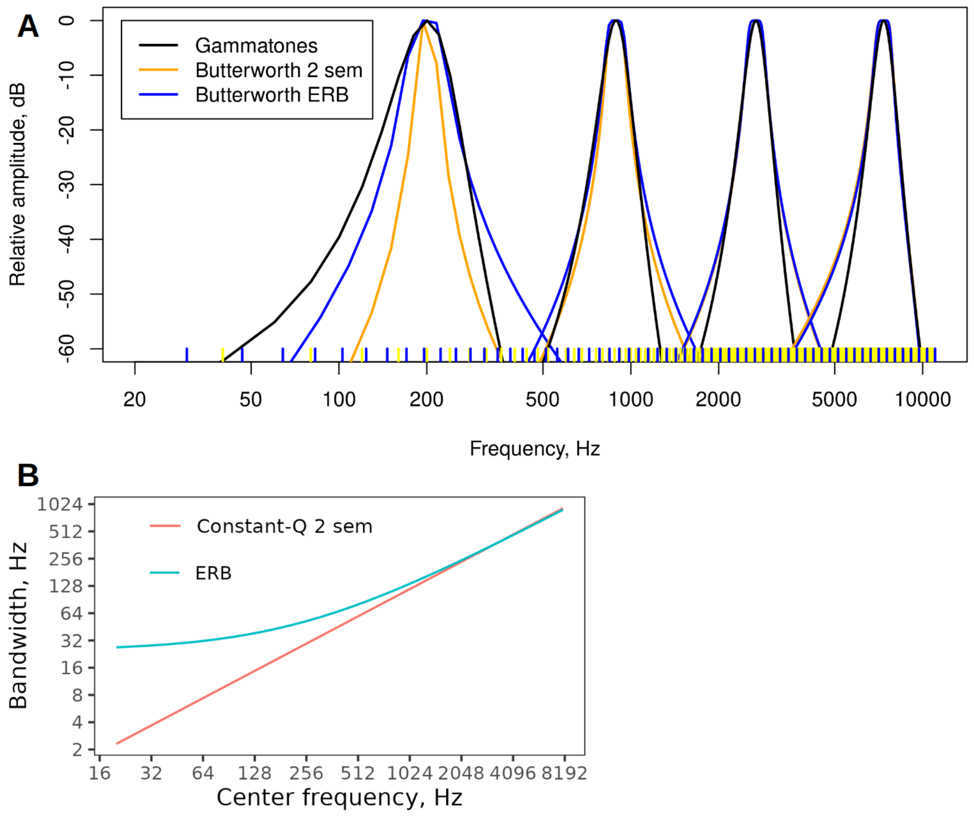

Fig. S3 A comparison of different auditory filters. (**A**) Frequency responses of Butterworth filters with ERB bandwidths (blue) are fairly similar to those of gammatones (black), and even constant-Q Butterworth filters with a bandwidth of two semitones (green) have comparable frequency responses, especially above ~500 Hz. Only four filters are shown to reduce clutter. The rug plots show bandwidths of an STFT spectrogram with a window length of 25 ms (green) and of a bank of 64 filters with ERB bandwidths (blue). (**B**) A comparison of constant-Q filters with a bandwidth of two semitones and filters with ERB bandwidth. ERB bandwidths are much larger in lower frequencies, but similar to the constant-Q filter above ~1 kHz.

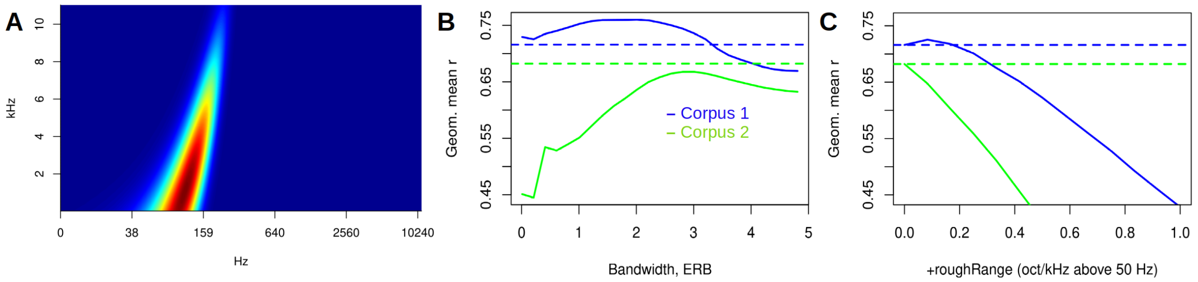

Fig. S4 Frequency-dependent weighting functions fail to improve roughness estimation. (**A**) The best identified multivariate normal weighting function: temporal dimension 110 ± 70 Hz, frequency dimension 1 ± 5 kHz, rho = 0.9, performance *r* = .74 for Corpus 1, .68 for Corpus 2, geometric mean .71. (**B**) Setting roughRange to a proportion of ERB bandwidth at the frequency of analyzed channel improves the results for Corpus 1 (blue), but not for Corpus 2 (green) relative to static cutoff points of 50 to 200 Hz (dotted lines, geometric mean *r* = .70). (**C**) Increasing roughRange from a baseline of (50, 200) for frequency channels above 50 Hz by a fixed number of octaves / kHz fails to improve performance. Tested on modulation spectra produced with 64 Butterworth filters with ERB bandwidths. See *optim_roughRange.html* for details.

The roughness range is theoretically expected to be positively associated with carrier frequency – thus, the range of modulation frequencies perceived as rough should be higher in high- vs. low-frequency bands (the top vs. bottom of a spectrogram). Indeed, the roughness zone of the average modulation spectrum of sounds weighted by their roughness ratings (i.e., the typical profile of a “rough” sound) appears to be leaning to the right in Fig. 2C. Several frequency-dependent weighting functions were therefore tested to check whether accounting for this phenomenon would improve the accuracy of roughness estimation. Indeed, a highly correlated multivariate normal weighting function with rho = .9 achieved slightly better overall performance (r = .71) than either simple cutoffs at (50, 200) Hz or a one-dimensional lognormal weighting function (r = .70 for both). However, this improvement is probably too slight to be of practical importance, and the robustness of multivariate weighting functions with respect to variation in the number and spacing of auditory filters would need to be verified. Two other frequency-adjusted roughness ranges were explored, with the range either given as a proportion of ERB bandwidth per filter or increasing log-linearly with frequency, but neither improved upon the performance of a static cutoff of (50, 200) Hz.

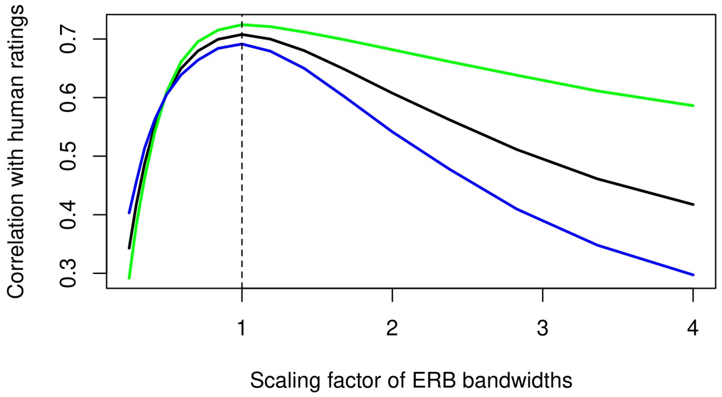

Fig. S5 Changing the scale of default ERB bandwidths does not improve the performance of Butterworth filters: the optimal correlation with human ratings is achieved when the scale factor = 1.

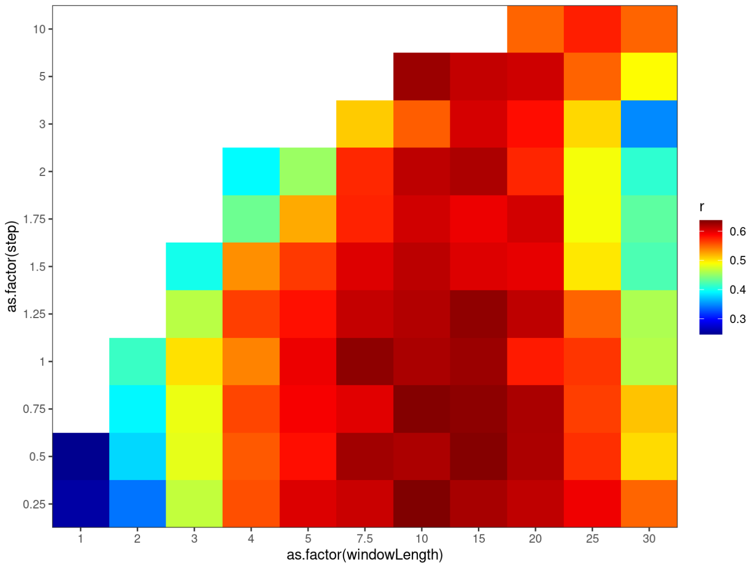

Fig. S6 Optimization of roughness estimation via 2D modulation spectra of STFT spectrograms and a lognormal(100, 8) weighting function. The best results are achieved with a window length of 10 ms and a step of 0.25 ms: r = .67 for Corpus 1, .61 for Corpus 2, geometric mean = .64. See *optim_STFT.html* for details.

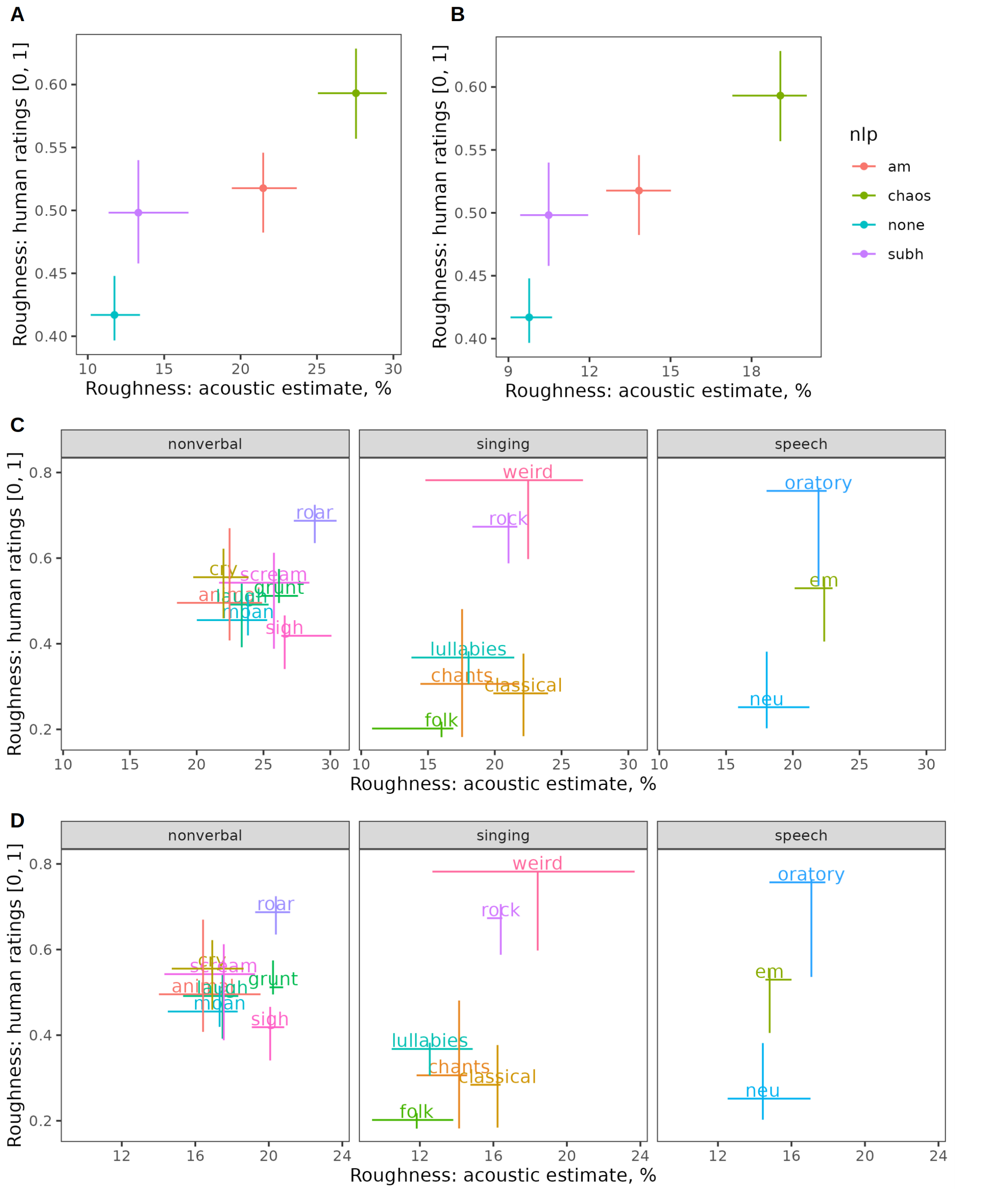

Fig. S6 A comparison of roughness per category in the two corpora when estimated by human raters vs. computer algorithms. (**A**) Corpus 2, auditory spectrogram. (**B**) Corpus 2, STFT. (**C**) Corpus 1, auditory spectrogram. (**D**) Corpus 1, STFT. The points are medians, and the bars show 50% interquartile ranges. *nlp* = nonlinear phenomena; *am* = amplitude modulation; *subh* = subharmonics (for details on corpus 1, see (Anikin et al., 2021)); *neu* = neutral speech; *em* = emotional speech (for details on corpus 2, see (Anikin et al., 2023)).

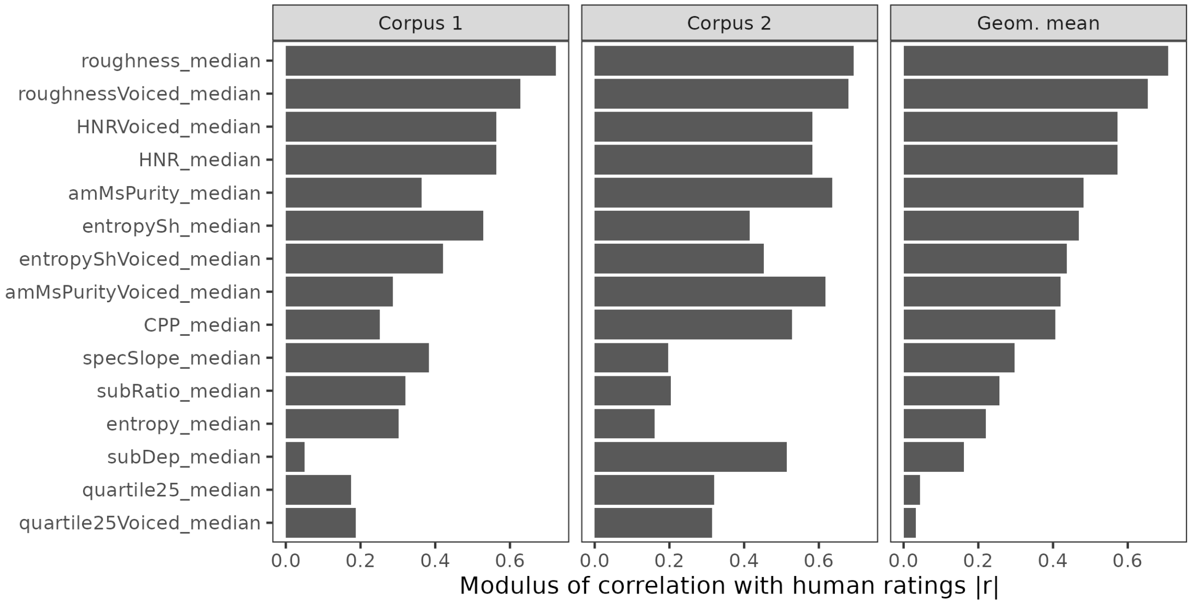

Fig. S7 Absolute values of Pearson's correlation between human roughness ratings and various descriptives returned by the *analyze()* function in *soundgen* library. Various spectral descriptives have been investigated in the context of estimating roughness (Barsties, Maryn, et al., 2018). Here, HNR, CPP, Shannon entropy of the spectrum, and measures of amplitude modulation do capture some of the perceptual quality of rough sounds and may be considered as partial proxies if proper roughness analysis cannot be performed. *HNR* = harmonics-to-noise ratio, *amMsPurity* = purity of amplitude modulation measured from the modulation spectrum, *entropySh* = Shannon entropy of the spectrum, *CPP* = cepstral peak prominence, *specSlope* = spectral slope, *subRatio* = the ratio of fundamental to subharmonic frequency, *entropy* = Wiener entropy of the spectrum, *subDep* = depth of subharmonics, *quartile25* = first quartile of spectrum. The *"Voiced"* variables are calculated only for the frames considered voiced. See *soundgen* documentation for details.

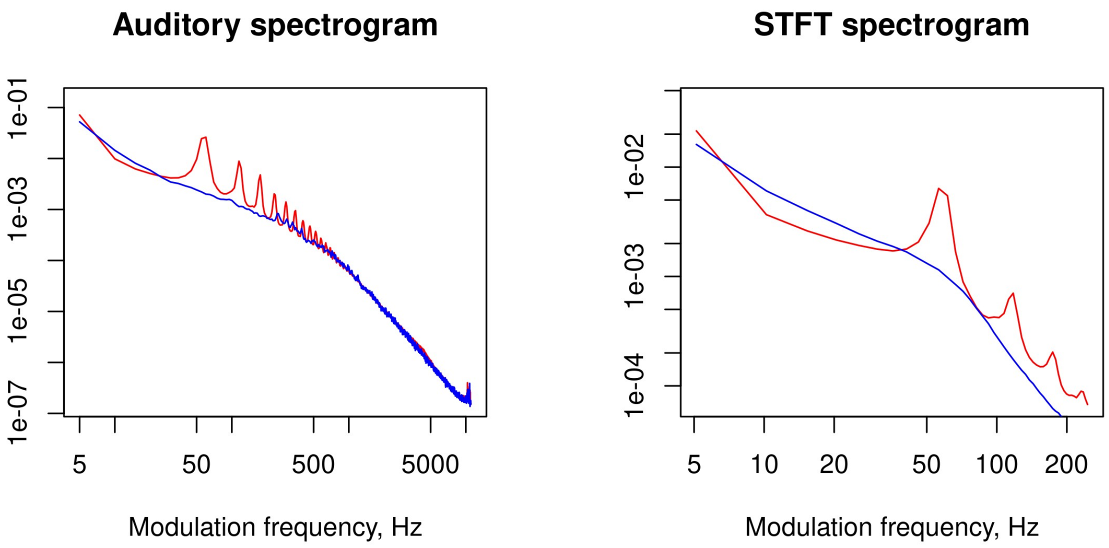

Fig. S8 Modulation spectra of two singing recordings from Corpus 2 whose perceptual ratings of roughness were the highest (red, file "206_singing_weird_M_throat_Huun-Huun-Tu2.wav") and lowest (blue, file "189_singing_folk_F_WestEur_Deanta.wav"), respectively. The rough recording is marked by significant peaks in the modulation spectrum between 50 and 200 Hz caused by strong subharmonics. The modulation spectra are averaged across all frequency bands in the spectrogram. Auditory spectrograms can resolve much higher modulation frequencies than STFT (up to half the sampling rate of the original audio), but this is not needed for roughness analysis.
